# Developing population interventions with migrant women for maternal-child health: a focused ethnography

**DOI:** 10.1186/1471-2458-13-471

**Published:** 2013-05-14

**Authors:** Anita J Gagnon, Franco Carnevale, Praem Mehta, Hélène Rousseau, Donna E Stewart

**Affiliations:** 1 Ingram School of Nursing, McGill University, 3506 University St., Room 207, Montreal QC H3A 2A7, Canada; 2 Department of Obstetrics and Gynaecology, McGill University, Montreal H3A 2A7, Canada; 3 McGill University Health Centre, Montreal QC H3H 2R9, Canada; 4 McGill University and CSSS de la Montagne 5700 Côte-des-Neiges Montreal QC H3T 2A8 Canada; 5 University of Toronto and University Health Network 200 Elizabeth St, EN-7-229 Toronto ON M5G 2C4 Canada

**Keywords:** Immigrants, Women, Health services, Qualitative research, Childbirth

## Abstract

**Background:**

Literature describing effective population interventions related to the pregnancy, birth, and post-birth care of international migrants, as defined by them, is scant. Hence, we sought to determine: 1) what processes are used by migrant women to respond to maternal-child health and psychosocial concerns during the early months and years after birth; 2) which of these enhance or impede their resiliency; and 3) which population interventions they suggest best respond to these concerns.

**Methods:**

Sixteen international migrant women living in Montreal or Toronto who had been identified in a previous study as having a high psychosocial-risk profile and subsequently classified as vulnerable or resilient based on indicators of mental health were recruited. Focused ethnography including in-depth interviews and participant observations were conducted. Data were analyzed thematically and as an integrated whole.

**Results:**

Migrant women drew on a wide range of coping strategies and resources to respond to maternal-child health and psychosocial concerns. Resilient and vulnerable mothers differed in their use of certain coping strategies. Social inclusion was identified as an overarching factor for enhancing resiliency by all study participants. Social processes and corresponding facilitators relating to social inclusion were identified by participants, with more social processes identified by the vulnerable group. Several interventions related to services were described which varied in type and quality; these were generally found to be effective. Participants identified several categories of interventions which they had used or would have liked to use and recommended improvements for and creation of some programs. The social determinants of health categories within which their suggestions fell included: income and social status, social support network, education, personal health practices and coping skills, healthy child development, and health services. Within each of these, the most common suggestions were related to creating supportive environments and building healthy public policy.

**Conclusions:**

A wealth of data was provided by participants on factors and processes related to the maternal-child health care of international migrants and associated population interventions. Our results offer a challenge to key stakeholders to improve existing interventions and create new ones based on the experiences and views of international migrant women themselves.

## Background

In 2010 there were an estimated 214 million migrants worldwide, half of whom were women [1]. Through World Health Assembly Resolution 61.17, ‘Health of Migrants’, the international community has identified migrant health as a priority, recognizing the health of migrants as a human right and calling for World Health Organization (WHO) Member States to promote migrant-sensitive health policies and programs [2]. The *Report of a Global Consultation on the Health of Migrants – the Way Forward* summarizes a consultation convened in response to the Resolution in which several priorities were identified including ensuring health systems are migrant-sensitive [3]. Given that international migrant women are responsible for an increasing number of births in receiving countries, their health and care and that of their infants’ are migrant issues meriting closer scrutiny. Examining these issues from the perspectives of migrant women themselves can provide data from which key stakeholders may act to promote migrant-sensitive maternal-child health care. In our project, we aimed to describe how international migrant women in Canada respond to health issues that arise in the months and years post-birth, and the related population interventions (i.e., policy, program, and resource distribution decisions) that they suggest merit revision or should be created to respond to their needs. In so doing, we sought to respond to the Resolution by promoting migrant-sensitive maternal-child health policies and programs.

### Population interventions affecting international migrant women

**Figure 1:**
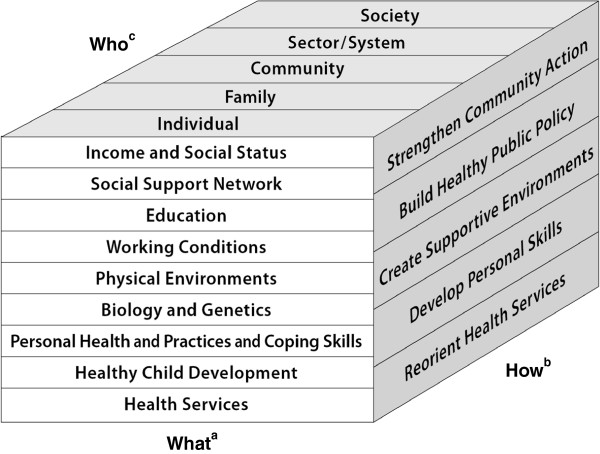
**Population Health Promotion Model [**[4]**]. **^a^On WHAT should we take action? Strategies for Population Health points out that action must be taken on the full range of health determinants (the WHAT). ^b^HOW should we take action? The Ottawa Charter on Health Promotion calls for a comprehensive set of action strategies to bring about the necessary change (the HOW). ^c^WITH WHOM should we act? Both documents affirm that, in order for change to be accomplished, action must be taken at various levels within society (the WHO).

Population interventions have been conceptualized in the Public Health Agency of Canada (PHAC)’s Population Health Promotion Model [4] (Figure 1). It guides action by asking three questions: “On *what* should we take action?” (i.e., broad range of health determinants [5]); “*How* should we take action?” (i.e., comprehensive action strategies); and “With *whom* should we take action?” (i.e., various levels of actors). Population health interventions include policies and programs that operate within and outside the health sector with the potential to impact health at a population level [6], and they encompass a range of social determinants of health [7]. Considering migrant women, two sectors are key - immigration and health [8]. Examples of interventions in the immigration sector are: access to political rights, immigration assistance programs, and access to essential language skills. Examples in the health sector include availability of interpreter services and type of national health system [9]. This Model offers a framework by which to examine population interventions with international migrant women.

Although migrants often have reduced entitlements in receiving countries and greater exposure to lack of employment and housing or poor working and living conditions, only one recent study has directly examined national policies and the perinatal health of migrant women. Bollini *et al.* used high or low naturalization rates to define receiving countries as having strong or weak integration policies and subsequently compared receiving-country and immigrant populations in these two country groupings using published data [10]. Compared to receiving-country women, immigrant women showed a clear disadvantage for all outcomes, however risks were significantly reduced in countries with strong integration policies. This study offers evidence of an effect of population interventions on perinatal health outcomes.

Assignment of migrants to immigration classes by receiving countries serves to operationalize population interventions applying to them. Within the heterogeneous migrant group, there are a number of distinct classes [11]: *immigrants* who generally choose to re-locate in a new country with the promise of a better life; *refugees*, forced to leave their countries to ensure their survival; *asylum-seekers*/*refugee claimants*, migrants who have formally requested the protection of a host country; *temporary residents* including workers, students and visitors; *undocumented*, those without a legal status in the host country, and *stateless*, those with no legal status in any country. Each of these classes is associated with regulatory restrictions related to eligibility for programs [12]. For example, *asylum-seekers* (also known as ‘refugee claimants’) in Canada have varied access to a federal government health insurance scheme (i.e., the Interim Federal Health Program IFHP) – a health insurance with restricted coverage based on source country, among other criteria [13]. *Immigrants*, on the other hand, do not have access to this federal insurance scheme, rather to provincial schemes offering coverage beyond emergency care and regardless of financial means but with time delays defined provincially. Access to employment, social benefits, and other programs also differ by immigration class. Hence, examining the relationship between immigration class and health is one approach to examining the relationship between population interventions and migrant health.

Studies of migrant perinatal and later post-birth health do not generally compare by immigration classes even though population-level responses to these issues will need to occur within immigration classes since this is where regulatory restrictions lie. Our previous Canadian studies are the exception [14-16]. In the recently completed study, the Childbearing Health And Related Service Needs of Newcomers (CHARSNN), we recruited 1127 women post-birth from one of eleven hospitals in Canada’s two main receiving-cities for migrants (Montreal and Toronto) [17]. At one week post-birth, *asylum-seeking* and *immigrant* women had greater risk for health problems than Canadian-born [OR_adj_ = 1.33 (95% CI 1.14, 1.55), 1.19 (1.04, 1.37), respectively] and all three migrant groups were more likely to have their problems unaddressed by the health care system [*refugees*, 2.27 (1.64, 3.11), *asylum-seekers*, 2.51 (1.93, 3.28), *immigrants*, 2.25 (1.73, 2.91)]. At four months post-birth, all migrant groups had greater risk of problems than Canadian-born [*refugees*, 1.91 (1.61, 2.27), *asylum-seekers*, 2.20 (1.89, 2.57), *immigrants*, 1.69 (1.46, 1.96)] and a greater likelihood that these were unaddressed [*refugees*, 2.18 (1.57, 3.02), *asylum-seekers*, 1.68 (1.28, 2.21), *immigrants*, 2.36 (1.75, 3.19)]. Further, the distribution of types of concerns differed by class, with asylum-seekers presenting the poorest health profile. These results provide evidence for the importance of analyzing perinatal and later post-birth migrant health by immigration class.

### International migrants and the health care system

The interface between international migrant women and the health care system is complex. There is evidence that migrants avoid accessing health care due to fear of harming their chances of settlement [18,19]. Factors such as language [20-26], transportation/geographic access [27,28], costs, and previous experience have been shown to influence access to health services [24,29-32]. Racism is less commonly reported but is also implicated as a barrier to access [18,33-35]. Language was a barrier reported by 15% of *immigrants* who identified problems in accessing health care in the Longitudinal Survey of Immigrants to Canada [36]. Difficulties for migrants to enroll with a family doctor have been reported [37] and this affects specialist care in Canada, since having a regular health care provider facilitates access to specialists [38]. Other studies have shown that once migrant women are ‘in’ the health care system, they are reluctant to follow professional recommendations for further care [16]; barriers to care have been found to be both personal and systemic [39,40]. Taken together, a range of factors – from both the individual and ‘system’ perspectives – play a role in health care access and use by migrant women.

One study of nurse contacts to asylum-seeking women with multiple health problems, found several structural barriers to receiving care [41]. These included: refusal to care for infants (born as Canadians) whose mothers were covered under Canada’s IFHP [41]; refusals to care for others covered under IFHP [42,43]; difficulties of public health nurses to reach women [41]; a general lack of assessment, support, and referral for psychosocial concerns [41]; isolation [41]; language barriers [41-43]; low health literacy [41]; and confusion and limitations of the IFHP [41-43]. Population interventions could well respond to several of these structural barriers.

Some studies have shown that once eligible for provincial health care insurance, patterns of healthcare utilization among migrants are largely determined by their length of time in Canada [29]. When international migrants first arrive, attending to their health is rarely a priority; employment and housing take precedence [19,44]. Migrants are less likely to visit a regular health care professional [45] and consequently are less likely to receive preventative and chronic care [46]. Their first medical consultation is usually in an emergency walk-in setting. Knowledge of the health care system reportedly comes from speaking with other migrants in their community [20,44,47]. In addition to suggesting the importance of considering length of time in country as a predictor of health care use by migrants, this body of literature points to the role of several population interventions in the immigration sector as having a role in health and timing of health care access.

### Resilience

Although studies have shown international migrant women to be at greater health risk and to have more difficulty accessing the health care system than non-migrants, there is simultaneously evidence of abundant strength in the face of adversity in these women. Empirical data and anecdotal reports from project nurses in the CHARSNN study drew our attention to the fact that some migrant women with high psycho-social risk profiles (e.g., histories of severe trauma experiences) at study entry were functioning well at the 1-week and 4-month contacts, suggesting that maternal resiliency is likely playing an important role in the maintenance of their health and that of their infant’s. "Resilience" has been defined as, “a dynamic process in which psychological, social, environmental and biological factors interact to make an individual, at any stage of life, develop, maintain or regain their mental health despite exposure to adversity” [48]. Adversity is defined as negative events that are associated with adjustment difficulties or subsequent mental disorders. These events include violence, war, traumatic events, poor parenting, poverty, or forced migration. Resilience research is based on a strengths model, rather than a deficit or problem-oriented approach. Better understanding of resilience has the potential to inform strategies of how best to support and protect individuals of all ages in adverse situations and to assist in designing interventions for those who have experienced adversity and developed mental health problems to regain resilience and lead productive lives [49-51]. The most recent resiliency research [50-53] emphasizes that resiliency fundamentally rests on relationships and is a process or phenomenon of positive adaptation despite adversity, and not simply a personal characteristic of an individual. Family, peers, schools, community, social supports and social policy are seen as vital contributors to resiliency. The CHARSNN database offered us the opportunity to identify resilient women from whom we could learn positive approaches to health challenges and beneficial population interventions. We sought to tap into the knowledge of these women in particular, as well as that of women with a similar profile who were functioning less well at follow-up in order to ultimately suggest optimal population interventions for consideration by key stakeholders.

### Summary

The international community has identified migrant health as a priority and with an increasing number of international migrants giving birth in receiving countries, their health takes on added importance. Population interventions in both the health and immigration sectors affect the health of migrant women around the time of birth and thereafter yet there is no study of which we are aware which gives voice to international migrant women’s experience of these interventions. Many international migrant women have lived through extremely difficult situations and yet have managed to maintain their health and that of their infants and as such are a group from whom we can learn much. We seek to begin to fill this knowledge void in the current focused ethnography. Our research questions are as follows:

1. What individual processes (e.g., actions, cognitions) are used by migrant women to respond to maternal-child health and psychosocial concerns during the early months and years after birth?

2. What contextual factors (e.g., physical, social, structural) and related processes enhance or impede their resiliency (i.e., strength to deal with, confront, and endure) to maternal-child health and psychosocial concerns?

3. What policy and/or program interventions are suggested by them and by key stakeholders to be effective or ineffective for maternal-child health and psychosocial concerns?

4. What nursing interventions are suggested by them and by key stakeholders to be effective or ineffective for maternal-child health and psychosocial concerns?

## Methods

### Design

Focused ethnography [54,55] including in-depth interviews [56-59] and participant observation [60,61] were conducted. Focused ethnographies are time-limited exploratory studies within a discrete group of persons or community [54,55]. Focused ethnography has been chosen over other methodologies due to the significant linguistic, cultural and social diversities of the populations we examined. Ethnography is highly recognized as a methodology for examining socio-cultural communities in their “natural” settings (i.e., in their homes, community settings), enabling the researcher to better understand social phenomena as they are practiced, not only as they are disclosed verbally.

### Setting and participants

Our study population was drawn from that of an earlier study, CHARSNN [14]. In that study, women speaking any of 13 languages [Arabic, Dari/Persian, English, French, Mandarin/Cantonese (oral; ‘simple’ and ‘complex’ Chinese written), Punjabi, Russian, Serbo-Croatian, Somali, Spanish, Tamil, and Urdu], together with their infants, were recruited from postpartum units of hospitals serving a high percentage of migrants to Canadian cities receiving the greatest number of refugees (Toronto, 32.8%; Montreal, 21%; and Vancouver, 7.7% [62,63]). Data on the index pregnancy, maternal/infant physical and psychosocial health, abuse/discrimination experienced, and services used were gathered. Those data were used to identify women for the current study. Sixteen international migrant women living in Montreal or Toronto who had participated in CHARSNN, had consented for additional contact, and had had a high psycho-social risk profile at baseline, were recruited to extend their participation to this project. A high psycho-social risk profile was defined as having a: family income < $10,000/yr. and having experienced violence or war or trauma (specifically, having originated from or spent time in an area of armed conflict *and/or* experienced/witnessed/heard any trauma events listed on the *Harvard Trauma Questionnaire* (*HTQ*) *and/or* had been physically abused in the last 12 months *and/or* had a pregnancy which was or may have been the result of forced sexual intercourse).

Having met the criteria for high psycho-social risk, women were then identified as being vulnerable or resilient for study purposes. Those defined as vulnerable had the following characteristics at 4 months post-birth: scored ≥10 on the *Edinburgh Postnatal Depression Scale* (*EPDS*) *and/or* presented symptoms of depression/anxiety/somatization [on the *Hopkins Symptom Check List* (*HSCL*)] *and/or* presented symptoms of Post-traumatic Stress Disorder [PTSD (on the *HTQ*)]. Resilient were those who: had a score <10 on the *EPDS*, no symptoms of depression/anxiety/somatization (on the *HSCL*), and no symptoms of PTSD (on the *HTQ*). Research assistants telephoned the CHARSNN participants who met the criteria to explain and seek their consent to extend their involvement to this study. Ethical approval from the McGill University Faculty of Medicine Institutional Review Board was obtained prior to seeking consent.

### Data collection and analyses

Focused ethnography [54,55] including in-depth interviews [56-59] and participant observations [60,61] were conducted by bi-lingual graduate student research assistants. The qualitative research leader (FC), an experienced qualitative researcher and educator, trained the research assistants to conduct these interviews and observations. Questions included in the in-depth interviews were driven by our interest to understand processes of care that are likely to, or clearly will not, work with migrant women and included the full range of experiences of early to later parenting (see Additional file 1). The interviews were carried out over two home visits and were audio-recorded if consent was provided. They were then transcribed [64] and coded/analyzed by the three authors (two investigators and a research assistant). Three observation periods were made per participant and were conducted at sites selected by the participants as likely to offer information relevant to the research questions. Sites included: participants’ homes, community centers, places of worship, ‘ethnic’ markets, clinics, and daycare centers. The time and duration of the observations were determined by the participants’ availability and interest and ranged from 1.5-3.5 hours per session. Field notes were taken during interviews and participant observations.

A variety of qualitative data were produced: formal interview transcripts in English, French and Spanish (the languages of the participants meeting our inclusion criteria; French and Spanish transcripts were translated to English), descriptive accounts from the observations, and field notes from the interview and participant-observations. These were reviewed immediately following their production so that inconsistencies could be further investigated in subsequent visits. Data were analyzed throughout data collection for all 16 participants. The first level of data analysis consisted of a line-by-line thematic analysis of all of the interview and observational data, resulting in a list of codes. These codes were arranged into meaningful categories through the use of a matrix (i.e., for Research Question 1, the matrix consisted of identified coping actions crossed with coping resources). The structure of the analysis was adapted for each question. Data were examined collectively and comparatively; the latter examined similarities and differences between participant subgroups that were distinguished as either resilient or vulnerable. Definitions for each of the categories reported below can be found in Additional file 2. Intermediate and advanced analyses were performed primarily by FC in collaboration with AG and PM, following recognized processes for focused ethnography [54,55,65], in order to examine all data as an integrated whole as well as to conduct comparative analyses between resilient and vulnerable participants. Interview and participant-observation data for each participant were integrated into “case syntheses” to permit in-depth within-case analysis. The aim of the analyses was to identify interventions that are desired or sought by participants and the processes that enable or impede their utilization.

## Results

A total of 16 participants were interviewed (8 Montreal; 8 Toronto). Participants were originally from Burundi, Columbia, Ghana, Guyana, Iraq, Mexico, Nigeria, Rwanda, Sri Lanka, St. Vincent, and Yugoslavia. Women were between the ages of 27–38 years, had been in Canada ≤ 8 years, and had between 1–4 children each.

The presentation of our results is structured according to our four research questions.

### Individual processes (e.g., actions, cognitions) used by international migrant women to respond to maternal-child health (MCH) and psychosocial concerns after birth

Personal coping was inferred from the data as a framework for analyses for Research Question 1; i.e., the general concepts of *coping strategies* and *coping resources* as well as all of the specific strategies and resources reported below were identified in the data, no *a priori* “coping framework” was imposed on it.

Generally, coping strategies and resources were highly inter-related. Details are provided in Table 1. Twenty-three specific coping strategies were identified as ways of dealing with the concerns international migrant women faced; these were clustered into six themes. Some coping strategies were widely described as relevant by all participants. These included: *parenting, venting, developing/maintaining relationships, social networking, seeking formal health care, seeking tangible resources, living with emotional distress, living with emotional wellbeing,* and *dealing with a difficulty on your own*.

*“Look at the people around us in the bus…they look serious, stressed out, they do not smile, if they talk, they talk quietly…it’s like they are not living. This is probably why depression and suicide rates are higher here in Canada, because people keep all their stress inside themselves…”* (Mexico, 35 yrs. old; 3 yrs. 5 mo. in Canada)

**Table 1:** **Strategies and resources identified by participants to cope with being an international migrant to Canada**^**1**^

**Coping strategies**	
*Developing/drawing on skills*	*Living with emotional state*
• Advancing own education	• Living with emotional distress
• Learning language	• Living with emotional wellbeing
• Managing resources strategically	
• Parenting	*Using relational strategies (formal and informal)*
	• Developing/maintaining relationships
*Seeking health care*	• Helping others
• Seeking formal health care	• Interacting with professional staff
• Seeking informal healthcare	• Social networking
	• Venting
*Using internal strategies*	*Drawing on other strategies*
• Appreciating the Canadian social context	• Obtaining permanent residency (PR)
• Attending church	• Preserving one’s culture
• Dealing with a difficulty on your own	• Seeking advice/information
• Improving self-awareness	• Seeking tangible resources
• Praying	
• Withdrawing	
**Coping resources**	
*Dispositional*	*Skills*
• Attitude towards others	• Child behaviour management skills
• Being discerning	• Information-seeking abilities
• Determination	• Host country language
• Optimism	
• Prior experience	*Social*
• Self confidence	• Friendship
• Spirituality/faith/God	• Kinship Perception of belonging
• Vigilance	• Perception of belonging
	
*Health*	*Tangible resources*
• Emotional health	• Education
• Physical health	• Employment
	• Food/housing/money/clothing/car
	• Permanent resident status/citizenship

^1^see Additional file 2 for definitions.

Some coping strategies were highlighted as more widely relevant by vulnerable participants, when compared with resilient participants. These included: *helping others, seeking advice/information, managing resources strategically, seeking informal healthcare, learning language, and withdrawing*. Examples of learning language and seeking advice/information are highlighted in the following quotes:

*“Two months after the second baby was born, I went to the night (French) classes but my husband couldn’t take care of the children by himself so I had to stop…I start to follow the courses because I thought I could help the children with their homework and studying.”* (Sri Lanka, 31 yrs. old; 4 yrs. in Canada)

*“I feel so limited, I feel like a primate…when I talk to my oldest son, I feel like a cockroach. He says words that I don’t understand, he speaks very fast when he is not in a good mood. He asks me, don’t you know what that is??…but when he is in a good mood, he is the one who helps me do my homework…”* (Mexico, 36 yrs. old; 5 yrs. 9 mo. in Canada)

Only one coping strategy was reported exclusively by resilient international migrant women - *preserving one’s culture* (e.g., speaking the languages of one's country of origin). The remaining strategies were reported by both groups of women. However, some coping strategies were identified only among a very small number of participants: *interacting with professional staff, appreciating the Canadian social context, obtaining PR* [permanent residence]*, improving self-awareness, and advancing own education.*

*“We have to get adapted (to living in Canada), whether we like it or hate it, we have to because the future of our kids, this is where it is. Now, it is much better than 7 months ago.”* (Iraq, 41 yrs. old; 1 yr. 9 mo. in Canada)

Twenty coping resources were reported by participants as either present or absent – facilitating or impeding their coping. These were clustered into five themes. Data were examined by comparing resilient versus vulnerable status; no differences in resources used were identified by group. All coping resources were reported as having been used by both groups of women.

The use of all remaining coping strategies (i.e., *attending church* and *praying*) and the presence or absence of related coping resources varied across participants, with no identifiable pattern of association with participants’ measured resilience. Three coping resources (*attitude towards others, prior experience, spirituality/faith/God*) affected coping favorably when they were present.

### Contextual factors (e.g., physical, social, structural), and related processes, that enhance or impede international migrant women’s resiliency (i.e., strength to deal with, confront, and endure) to MCH and psychosocial concerns

*Social Inclusion* was identified as an overarching concern for all study participants. Processes and facilitators to inclusion are given in Table 2. Fourteen specific social processes were related to participants’ social inclusion, corresponding with 17 categories of facilitators for these processes. These processes to inclusion were not clustered into larger themes. Facilitators were clustered into three larger themes. Participants described facilitators as either being present or absent. When absent, they functioned as barriers to social inclusion.

*“Traveling all the way here, no father, no mother, no brother, no sister, it’s overwhelming, it’s scary…at the beginning was really scary.”* (Nigeria, 37 yrs. old; 8 yrs. in Canada)

*“And also, something else that really shocked me…I didn’t really have his support. Because when I told him, he was sleeping. He said, oh it’s fine, I think that’s normal. I said no I’m going to go to the hospital; you have to accompany me there. No, you go, I want you to go and then after I’ll meet you there. So that was the first thing that I found, I felt really alone.”* (Mexico, 36 yrs. old; 5 yrs. 9 mo. in Canada)

**Table 2:** **Processes and facilitators identified by participants for inclusion of international migrants in Canada**^**1**^

**Social inclusion processes**	
• Accessing education	
• Accessing food	
• Accessing health care (formal and informal)	
• Accessing housing	
• Accessing work	
• Achieving a sense of financial security	
• Achieving a sense of kinship	
• Adapting to climate	
• Attending to unspecified activities	
• Fostering parenthood	
• Overcoming language barriers	
• Perceiving safety	
• Perceiving security	
• Pursuing personal interests	
**Facilitators to inclusion**	
*Financial*	*Other facilitators*
• Financial resources	• Adequate time
• Financial sufficiency	• Attainable job requirements
	• Childcare
*Social*	• Equitable laws/application of equitable laws
• Accompaniment	• Information/advice/counseling availability
• Appealing features of new culture	• Interpretation/translation
• Encouragement	• Manageable climate conditions
• Family help	• Permanent resident status/citizenship
• Favorable social environment	
• Friendship	
• Like community	

^1^see Additional file 2 for definitions.

A rich inter-relation was observed between these facilitators and social inclusion processes; i.e., multiple facilitators were related to most processes. Social inclusion involves multiple processes, each fostered by a wide range of factors.

Some social inclusion processes were widely recognized as relevant by all participants, regardless of their measured resilience. These included: *perceiving safety, achieving a sense of financial security, achieving a sense of kinship*, *accessing formal health care*, *fostering parenthood*, and *attending to non-specified activities*. An example for achieving a sense of kinship can be found in what this woman from Serbia had to say:

*“People who come here they cry like every day… because they never go out from Serbia, well I know some people they never go out from Serbia and now they come far away here, they know nobody, because I was lucky I has my family here, and this way maybe I don’t feel that…I have family that is long time here, and then help that has friend and you know?” (*Serbia, 32 yrs. old; 6 yrs., 7 mo. in Canada)

However, some processes to inclusion were identified only among a very small number of participants: *adapting to climate, pursuing personal interests, perceiving security, accessing food,* and *accessing housing.* Some inclusion processes were highlighted as more widely relevant by vulnerable participants, when compared with resilient participants. These included: *overcoming language barriers, accessing work*, *accessing informal health care*, and *accessing education*. All facilitators appeared more “idiosyncratic”; i.e., they were reported variably across participants with no identifiable pattern of association with participants’ measured resilience.

### Policy and/or program interventions suggested by international migrant women as being effective or ineffective for MCH and psychosocial concerns

Data were examined to identify policy and/or program interventions that participants regarded as effective or ineffective. These are listed in Tables 3 and 4. Eleven categories of interventions were described by participants. These interventions included: daycare, education, employment, food support, health care (e.g., clinics, hospitals, medical insurance, nurses, physicians), housing support (e.g., general, shelters, YMCA), immigration procedures, organizations/programs (e.g., church, CLSC/community health center, family home visitor, mixed, OLO, PRAIDA, SARIMM), psychosocial care (psychologist, social worker), social groups/activities, and welfare/government financial assistance. Participants described seven types of services provided by these programs and reported on the quality of service related to each intervention which included a balance of positive and negative accounts.

**Table 3:** **Interventions and types and quality of services used post-birth by international migrants to Canada**^**1**^

**Interventions**	
• Daycare	**Types of services** (provided within the interventions/programs)
• Education	• Child care and development
• Employment	• Information/advice
• Food support	• Maternal care
• Health care (e.g., clinic, hospitals, medical insurance, nurses, physicians)	• Non-specified services
• Housing support (e.g., general, shelters, YMCA)	• Provision of tangible items
• Immigration procedures	• Referral
• Organizations/programs (e.g., church, CLSC/community health center, family home visitor, mixed, OLO, PRAIDA, SARIMM)	• Teaching
• Psychosocial care (psychologist, social worker)	**Qualities of services** (within the interventions/programs)
• Social groups/activities	• Access
• Welfare/government financial assistance	• Affordability
	• Attitude
	• Availability
	• Satisfaction

^1^see Additional file 2 for definitions.

**Table 4:** Modifications suggested by participants to current, and suggestions for future, programs for international migrants post-birth

**Interventions**	**Suggested modifications**
Access to housing/Settlement services	• Extra services should be provided by settlement agencies
Education	• More afterschool/homework programs for working parents
	• Teachers should be more involved with students around bullying because parents can’t be at school
Employment	• Wants opportunity to start somewhere - wants her skills to be known
	• Have a liaison to local organizations that are hiring; a person to guide and focus newcomer careers to Canadian opportunities
Family home visitor	• Volunteer could come sit a few minutes to care for child and relieve mother for a few minutes, allow her to sleep, adjust to life as newcomer
	• Home visits for all mothers in first few years after birth to see how mother is coping
Health care: professionals	• Transition is hard on couples, service to help them support each other – psychologist to help individuals and couples
Social groups/activities	• Would like social groups and activities with and without children to meet other parents so they could share their experiences
	• More community activity centers
	• CACI could offer group meetings to new immigrants
Other	• Center where all information and support is provided under one roof
	• More support for undocumented immigrants
	• Have someone to research an issue (e.g., passport) which can lift a burden for a newcomer who does not know where to start

**Figure 2Figure 2:**
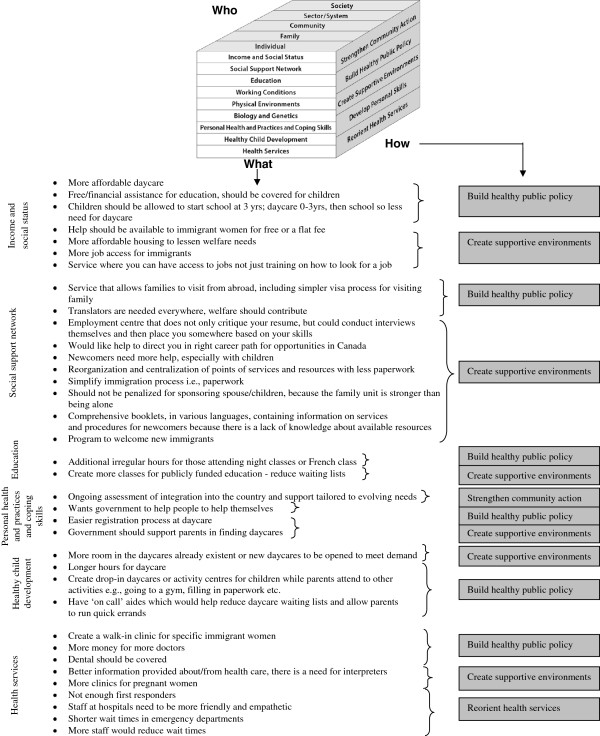
Suggested intervention modifications within education/daycare, housing, employment, immigration, welfare, and health sectors in the model.

Vulnerable mothers described referral as a type of service in exclusively positive terms. Both vulnerable and resilient mothers described information/advice, teaching, child care and development, and provision of tangible items in more positive terms than negative. Participants also suggested modifications and creation of new programs that they considered necessary. These programs were categorized according to the Population Health Promotion Model [4]. Each suggestion was placed into the corresponding level of *what, how,* and *who* suggested by the cubed model. Those programs that were deemed to fit the ‘who’ level of ‘sector/system’ have been presented in Figure 2. The remaining suggestions can be found in Table 4.

### Nursing interventions suggested by international migrant women to be effective or ineffective for MCH and psychosocial concerns

Data were examined to identify nursing interventions that participants regarded as effective or ineffective. These are detailed in Table 5. Four sites of nursing service provision were identified. Participants described five types of services provided at these sites, and their satisfaction with the nursing service they received in three aspects. Overall, nursing services were consistently described as effective, with some critical accounts regarding attitude and availability reported by vulnerable participants. Table 6 gives modifications suggested by participants for a range of nursing services.

**Table 5:** Type and quality of nursing services used post-birth by international migrant women in Canada

**Sites**	
• CLSC/clinic	**Types of services**
• Home	• Assessment of baby
• Hospital	• Information/advice
• Info-Santé/Tele-health	• Maternal care
	• Referral
	• Teaching
	**Qualities of services**
	• Attitude
	• Availability
	• Satisfaction

^1^see Additional file 2 for definitions.

**Table 6:** Modifications suggested by participants to post-birth nursing services for international migrants in Canada

**Types/qualities of nursing services**	**Suggested modifications**
Availability	• More funding for nursing/health fields
	• More public health nurses to visit new moms
Maternal care	• Mothers to have a nurse to help for at least a month postpartum
	• Nurses should assess how the mother is coping
	• CLSC nurses should ask about husband’s role
Teaching	• Nurses to help with breastfeeding, especially for mothers who had C-sections
Information/advice	• Nurses should be available to answer questions
	• Would like service to access interpreter
	• Language improvement needed in nursing services - essential for better communication
Assessment of baby	• Nurses should assist in taking care of baby
	• More follow-up visits to check health of newborn

^1^see Additional file 2 for definitions.

## Discussion

Each of the four research questions looked at a different aspect of a new immigrant mother’s health and social concerns around the time of birth. For research question 1, while looking at data about individual processes used to respond to maternal-child health, the overarching themes that emerged were coping strategies and the resources that allow these mothers to adapt to a new life in Canada. Some coping strategies such as: *seeking formal health care, seeking tangible resources,* and *dealing with a difficulty on your own,* were found in both groups of women leading us to understand that whether a woman was vulnerable or resilient, she turned to the same resources to cope in a new country. As can be seen from the literature, seeking formal health care is often a last resort for any new migrant to Canada [19,29,44]. A number of barriers namely health insurance, time off work, and childcare prevent new migrant mothers from seeking formal health care not just for themselves, but in many instances for their children as well. Type or lack of health insurance have been previously reported in the literature as barriers [41-43]. Seeking tangible resources refers to the international migrants’ ability to access adequate housing, clothing, and a car in addition to many other things necessary to start life again in a new place and the data showed us that being able to access these tangible things gives one a sense of belonging to a new country. The need to deal with a difficulty on your own refers to a lack of support from those outside the immediate family circle, either from the community or from other relatives. However, it can also make some international migrants stronger for having to take the initiative to find where to go for help and how to deal with any unknown situation that may arise.

One coping strategy was found to be used primarily by the resilient group: *maintaining culture*. This strategy entailed things such as speaking one’s mother-tongue at home with their children or eating foods from their country of origin. Maintaining one’s culture often gives international migrants a sense of security and belonging to something. They may not necessarily feel like they belong in the new country, but they may feel comfortable in their own community away from home making it easier for them to cope with life here.

For research question 2, while looking at data about contextual processes used to respond to maternal-child health, the overarching theme that emerged was social inclusion. Certain social inclusion processes were widely recognized as relevant by all participants including: *perceived safety, achieving a sense of financial security, achieving a sense of kinship*, *accessing formal health care*, *fostering parenthood*, and *attending to non-specified activities*. Some inclusion processes were highlighted as more widely relevant by vulnerable participants including *overcoming language barriers, accessing work*, *accessing informal health care*, and *accessing education*. Among these issues, language barriers have been most frequently reported in the literature [20-26,36].

The use of focused ethnography, through combined interview and participant-observation data collection methods, helped identify some complex overarching findings. Although some distinctions were identified between vulnerable and resilient participants, these did not convey a clear pattern of discernment between the groups. Moreover, when interview and participant-observation data for each participant were integrated into case syntheses for within-case analysis, participants who had been identified as vulnerable demonstrated significant actions, capacities, and resources in their management of the challenges they faced. Vulnerable participants were much more resilient than their measured categorization might imply. This raises important implications for how resilience measures are interpreted.

Moreover, our analysis of the case syntheses also highlighted the complex interplay between findings for research questions 1 and 2. The first question focused on intra-personal perspectives, while the second question examined contextual considerations. This focused ethnography highlighted that resilience among migrant mothers needs to be understood as a person-environment interaction phenomenon. Each mother will draw on her own personal capacities to cope, which will be enabled or impeded depending on the environment within which she is coping. Resilience and vulnerability are not exclusively indications of personal strengths or deficits. Rather, relatively resilient mothers can be made vulnerable by unfavorable environments. Conversely, the health and social inclusion of relatively vulnerable mothers can be fostered by favorable environments. This is analogous to a phenomenon identified in disability studies. Disability is rooted in the interplay between personal capacities and physical and social barriers in the environment: environments are enabling or disabling [66].

For research questions 3 and 4, participants provided specific feedback on interventions, type and quality of services provided within interventions, and they suggested modifications. For the vast majority, their feedback could be categorized within known social determinants of health lending further credence to the importance of sectors outside of the health system to migrant women’s health.

Our study is not devoid of limitations. Our definitions of vulnerable and resilient may be challenged. We were unaware of any generally agreed-upon definitions of these terms hence we chose to operationally define them using measures of mental health at our disposal. Our access to observation sites was occasionally restricted, especially at clinics and daycare centers, because employees at these locations felt that being observed was equivalent to being evaluated hence we were only able to make limited observations in those settings.

## Conclusions

A wealth of data was provided by participants on factors and processes related to the maternal-child health care of international migrants and their associated population interventions. Among the six thematic categories of coping strategies identified by participants, several strategies were reported predominantly by vulnerable women, one was described by resilient participants, while remaining strategies were identified among both groups. Five categories of coping resources were reported by participants as either present or absent – facilitating or impeding their coping. All coping resources were reported as having been used by both resilient and vulnerable groups of women, although some were more prominent in one group or another.

Social inclusion was identified as an overarching concern for all study participants. Fourteen specific social processes were related to participants’ social inclusion, corresponding with seventeen categories of facilitators for these processes; processes and facilitators were inter-related. Some processes were highlighted as more widely relevant by vulnerable participants, while others were reported variably across participants with no identifiable pattern of association with participants’ measured resilience.

Although some distinctions were identified between vulnerable and resilient participants, participants identified as vulnerable demonstrated significant actions, capacities, and resources in their management of the challenges they faced. Vulnerable participants were much more resilient than implied by their measured categorization. A complex interplay was found between intra-personal and contextual perspectives. Resilience among migrant mothers needs to be understood as a person-environment interaction phenomenon. Resilient mothers can be made vulnerable by unfavorable environments, while the health and social inclusion of relatively vulnerable mothers can be fostered by favorable environments.

In examining policy and/or program interventions that participants regarded as effective or ineffective, eleven categories of interventions were identified. Seven types of services were provided by these programs. Participants reported a balance of positive and negative accounts regarding the quality of service for each intervention. Overall, both vulnerable and resilient mothers described most services in more positive terms than negative. Several recommended modifications to existing programs or the needed creation of new programs were identified.

Participants identified nursing interventions that they regarded as effective or ineffective. Participants described five types of services provided in four sites of care, along with their satisfaction with the nursing service they received. In general, nursing services were described as effective, with some critical accounts regarding attitude and availability reported by vulnerable participants.

The experiences and views of international migrant women reflected in our results offer a challenge to several sectors of society to create or improve population interventions affecting the maternal-child health of migrants. With well over 100 million international migrant women worldwide [1], and given that they are responsible for an increasing number of births in receiving countries, taking action on behalf of their health and that of their infants is an imperative. By promoting migrant-sensitive policies and programs for maternal-child health, we will be directly responding to the international call for prioritizing migrant health stated in World Health Assembly Resolution 61.17, ‘Health of Migrants’ [2].

## Competing interests

The authors declare that they have no competing interests.

## Authors’ contributions

AG conceived of the study, participated in its design, obtained funding, had responsibility of general study oversight, contributed to the analyses, and drafted portions of the manuscript. FC participated in the study design, was responsible for all the analyses, and drafted portions of the manuscript. PM was responsible for overseeing data collection and preliminary data analyses, and drafted portions of the manuscript. HR and DES participated in the study design and revised the draft manuscript. All authors read and approved the final manuscript.

## Authors’ information

AG is co-leader of the international research collaboration, ROAM (Reproductive Outcomes And Migration), and coordinator of the Global Health Studies section of the Master’s program in nursing at McGill University. FC is currently a senior professor in qualitative methods at McGill University School of Nursing and a founding member of the McGill Qualitative Health Research Group. PM is research assistant in the Migration and Reproductive Health Research program with considerable experience in supervising and supporting a range of types of data collection. HR is Assistant Professor in the Department of Family Medicine and Assistant Dean of Rural Education at McGill University. DS is a University Professor in the Faculty of Medicine at University of Toronto and a Senior Scientist and Director of Women's Health at University Health Network in Toronto.

## Pre-publication history

The pre-publication history for this paper can be accessed here:

http://www.biomedcentral.com/1471-2458/13/471/prepub

## Supplementary Material

Additional file 1 Interview Questions. Click here for file

Additional file 2 Definitions/Descriptions of Codes. Click here for file
