# Supplementary material for: Developing population interventions with migrant women for maternal-child health: a focused ethnography
Source: BMC Public Health. 2013 May 14;13:471. doi: 10.1186/1471-2458-13-471 (PMC3733625; doi:10.1186/1471-2458-13-471)
Supplement: Additional file 1 — Interview Questions. [file 1471-2458-13-471-S1.pdf]

Additional File 1: Interview Questions

Research questions:
1) What processes do international migrants use during the early months and years after birth, and post-birth to respond to maternal-child health & psychosocial concerns?
2) What processes maintain or enhance resiliency to mother-child health and psychosocial concerns in international migrant populations?
3) What policy and/or program interventions are hypothesized by international migrant populations and key stakeholders to be effective for maternal-child health and psychosocial concerns?
4) What nursing interventions are hypothesized by international migrant populations and key stakeholders to be effective for maternal-child health and psychosocial concerns?

Preliminary Interview Guide

I would like to begin by telling you that I am going to ask you to talk about you and your family's usual activities and experiences. There are no right or wrong answers to the questions I am going to ask and you may choose not to answer some of the questions if you prefer. The questions are meant for us to learn about women new to Canada, understand more about how they take care of their and their families' health and other needs here in Canada, and how their communities might be involved with this. Your participation is precious and will allow us to develop programs and policies, adapted for immigrant mothers and their families. We will summarize the information you and other women give us and share the summaries with people who are responsible for developing programs and policies. ALL of your individual answers will be kept private, will only be used by the research team and no identifying information will be shared with immigration authorities.

Do you have any questions before we begin?

(Interview #1)

1. Summarize the participant's background information and ask them to clarify or correct any information if needed. Any changes since then?
   Background information will comprise data collected in an earlier study: Age, length of time in Canada, country of birth/ lived most as a child, migration trajectory, immigration status, marital status, number of children and name of the nurse from the home visit.
   (Verify data and enter in table format. Do not code)

2. Why did you move here (Canada, Montreal/Toronto)? Explain who made the decision. How was the decision made and what influenced it? (Probe: Decision maker and factors influencing the decision?)
   (Enter in table format. Do not code)

3. What is it like for you to be a newcomer in Canada?
   In Canada, have you found communities of people from the same country, culture or interest as you (groups, social activities, alliances) you feel comfortable being a part of? What about them made you feel comfortable?

4. Do you feel at home in Canada? (Yes/No)
   If yes, when did you begin to feel at home in Canada? What helps you feel at home?
   If no, what makes it hard to feel at home here?

5. When you **first had a child** in Canada, what types of challenges did you face? What personal qualities helped you deal with these challenges? (Probe: Inside you - determination, self confidence, sense of optimism, perseverance, faith, past experiences). How did you respond to these challenges? (Probe: family, friends, school, church, work, medical, nursing or social services, etc).
   For example:
   - For practical support e.g. food, clothing, car seat (what kind of practical support)?
   - If you were sad?
   - If you were physically unwell?
   - If you felt overwhelmed?

- If you felt isolated?
- If you needed health advice?

If they were they helpful to you, how?
If they were not helpful to you, why not?

6. In the **first years after giving birth** in Canada, what types of challenges did you face? What personal qualities helped you deal with these challenges? (Probe: Inside you: determination, self confidence, sense of optimism, perseverance, faith, past experiences). How did you respond to these challenges? (Probe: family, friends, school, church, work, medical, nursing or social services, etc).
   For example:
   - For practical support e.g. food, clothing, car seat (what kind of practical support)?
   - If you were sad?
   - If you were physically unwell?
   - If you felt overwhelmed?
   - If you felt isolated?
   - If you needed health advice?

   If they were they helpful to you, how?
   If they were not helpful to you, why not?

7. What, if anything, limited you from dealing with the challenges you faced since your child was born (internal e.g. fear, shyness/external e.g. money)?

8. Were there services that you wished you had had when:
   1) You first arrived in Canada?
   2) When you left the hospital with your baby?

   Are there services you wish you had now?

9. Sometimes people find experiencing difficulties can change their lives in different ways and in the end have a positive result. Did this happen to you? How?
   (Probe: new strength, new knowledge, more courage, change friends, leave partner, immigrate etc.)

10. Is there anything more you would like to share with me?

Thank you for taking the time to share your experiences and answer these questions. The information you gave is very helpful. Can we set up a time for the observation period? *(See list of possible places)*

11. What is the best way to reach you now? In the future?

Email: ______________________________________

Home phone (mother's): ________________________

Cell phone (mother's): __________________________

Friend's email: ________________________________

Friend's home phone: ___________________________

Friend's cell phone: ____________________________

(Interview # 2)
I really appreciate the time you gave me for our last interview. Is there anything you would like to add or share with me from our previous interview? (*Follow-up on any questions from previous interview or clarification on any answers you may have received*)

12. Going back to when you were first pregnant in Canada, tell me about **<u>any</u>** services or assistance you received that you liked? What did you like about it? Why?
    Tell me about any services or assistance you received that you disliked? What did you dislike about them? Why?
    What would you change to make these services better in serving mothers and their children?

13. Going back to when you were first pregnant in Canada, tell me about **<u>any</u>** nursing services or assistance you received that you liked? What did you like about it? Why?
    Tell me about any nursing services or assistance you received that you disliked? What did you dislike about them? Why?
    What would you change to make these nursing services better in serving mothers and their children?

14. What service or assistance provided **<u>specifically</u>** by nurses has been of help for you, your child or your family? Why?

15. Can you describe any difficulties **you** had in using a service? How did you respond to these difficulties? Were you able to overcome them? Please explain.

You mentioned services that you or your family used:

16. If you could change any service, which ones would you change? How would you change them? Please explain.

17. If you could create a service, please describe it as best as you can.
    (*Note: Look at the cube diagram in the reading from the Public Health Agency website that was in your RA package*)

18. If you could speak directly to those responsible for developing or changing income and social status, social support network, education, working conditions, physical environments, biology and genetics, personal health and practises and coping skills, health child development or health services, what would you tell them?

19. Is there anything more you would like to share with me?

This is the end of the interview.
Thank you for taking the time to share your experiences and answer these questions. The information you gave is very helpful. The research team will analyze the information you and others have provided and prepare summary reports for organizations and policy makers that work with migrants and new mothers. We would be happy to send you a copy of the report if you are interested in receiving one.

20. Would you like one? Yes/No

21. Would you be interested in meeting with other women who have completed this study to discuss the results? Yes/No

22. What is the best way to reach you? In the future?
