# Supplementary material for: Developing population interventions with migrant women for maternal-child health: a focused ethnography
Source: BMC Public Health. 2013 May 14;13:471. doi: 10.1186/1471-2458-13-471 (PMC3733625; doi:10.1186/1471-2458-13-471)
Supplement: Additional file 2 — Definitions/Descriptions of Codes. [file 1471-2458-13-471-S2.pdf]

Additional File 2: Definitions/ Descriptions of Codes

TABLE 1

Coping Strategies

Developing/ drawing on skills

Advancing own education: attending school or classes to learn skills for future employment or personal growth including mental health

Learning language: attending school or using other resources to learn the local language (English in Toronto or French in Montréal) and the ability to communicate in one of these languages

Managing resources strategically: skilful use of available resources such as money, time, and friends

Parenting: skills and actions of participants to promote their child's wellbeing

Seeking health care

Seeking formal health care: accessing professional medical and clinical services

Seeking informal health care: following medical advice or treatment from friends and family

Using internal strategies

Appreciating the Canadian social context: finding satisfaction with life in Canada

Attending church: keeping the memory alive of deceased family member or connecting with God by going to church

Dealing with a difficulty on your own: coping with a challenging situation by oneself

Improving self-awareness: experience of moving to a new country that leads to one growing as a person

Praying: praying to a higher being

Withdrawing: detachment of oneself from or avoidance of an uncomfortable situation

Living with emotional state

Living with emotional distress: negative emotions as a result of the stresses of moving to a new country and culture

Living with emotional wellbeing: positive emotions related to being in a new country and having family support

Using relational strategies (formal and informal)

Developing/maintaining relationships: building interpersonal relationships with friends and family

Helping Others: helping family members or others in the community gain information and/or overcome difficulties

Interacting with professional staff: any exchange between the participant and a person providing a service

Social Networking: making connections with people through the greater community (work, community centers, church etc.)

"Venting" with others (talking, crying, hitting): seeking comfort through emotional expression

<u>Drawing on other strategies</u>

Obtaining Permanent Residency (PR): emotions and processes related to obtaining Permanent Residency

Preserving one's culture: speaking the language or eating food from one's country of origin in order to maintain one's culture

Seeking advice/ information: Asking for help or looking for information on a variety of topics from people and the internet

Seeking tangible resources: acquiring resources that add to one's quality of life e.g. immigration status, housing, employment

Coping Resources

<u>Dispositional</u>

Attitude towards others: way a participant interacts with others

Being discerning: having good judgment about interacting with other people

Determination: having the drive to accomplish a task

Optimism: looking at the favourable side of a situation

Prior experience: Using prior situations and outcomes to deal with or improve one's current situation

Self confidence: having the ability and courage to handle unpleasant situations and act on one's own to accomplish something

Spirituality/faith/God: belief in a higher being or using this belief to deal with a situation

Vigilance: Paying attention to the needs of her child and being prepared and watchful about any situation that may arise or has arisen.

<u>Health</u>

Emotional health: relating to emotions and the mental health of a participant, how it affects their ability to function and what steps they take to overcome these feelings.

Physical health: relating to the physical wellbeing of a participant and issues around accessing health care professionals.

Skills

Child behaviour management skills: tactics used by a participant to manage her child's behaviour

Information-seeking abilities: the ability to look for information to solve a problem or orient oneself

Host-country language: relating to the knowledge of or lack of the local language (English in Toronto or French in Montréal), the ability to communicate in one of these languages and maintaining ties to home country by speaking native language

Social

Friendship: turning to friend or making friends to accomplish a variety of tasks: childcare, belonging to a community, and emotional wellbeing

Kinship: turning to family for support and help with childcare, emotional wellbeing and the relationship between family members.

Perception of belonging: feeling as though she is part of Canadian society and/or her immediate community.

Tangible resources

Education: having access to school to learn a language, to gain training for employment or to learn skills needed to be a better parent.

Employment: Anything related to finding work or working conditions in Canada

Food/ housing/ money/ clothing/ car: access to food, housing, money, clothing, or a car and how they are used to adapt to life in Canada

Permanent residence status/ citizenship: Issues pertaining to an individual's immigration status in Canada, the reasons for immigrating, and consequences

Table 2

Social inclusion processes

Accessing education: access to schools for mother and management of time and tasks around school.

Accessing food: access to food for newcomers through food banks and churches

Accessing health care
Accessing formal health care: accessing and using hospitals, clinics and health care professionals;

Accessing informal health care: accessing and using friends, family, and others as a source of information for medical care including birth and infant care

Accessing housing: access to any form of shelter (hotel, family accommodation, community shelter)

Accessing work: Anything related to finding work or working conditions in Canada

Achieving financial security: ways of accessing money (family, employment, government assistance) and the toll of not having enough money

Achieving sense of kinship: turning to family for support and emotional wellbeing as well as the relationship between family members

Adapting to climate: ability to adjust to the winters in Canada

Attending to unspecified activities: having free time to attend to other activities that haven't been specifically mentioned by the participant

Fostering parenthood: skills used by- and the actions of participants to ensure their child's happiness and wellbeing

Overcoming language barriers: ways used to deal with a lack of knowledge of the local language (English in Toronto or French in Montréal) and limitations of not knowing the language

Perceiving safety: relating to a participant feeling physically safe in different settings.

Perceiving security: having a sense of freedom from risk, danger, or anxiety based mainly on immigration status and other factors

Pursuing personal interests: activities the participant pursues for her own enjoyment and making time for these

Facilitators to inclusion

<u>Financial</u>
Financial resources: accessing money to fulfill a variety of needs

Financial sufficiency: having enough money to fulfill one's needs

Social

Accompaniment: having someone physically present to help translate, to share experiences with, and not feel isolated

Appealing features of new culture: of or relating to the impact of a new culture on the participant or how their own culture can be maintained

Encouragement: encouragement from others to pursue something or how situations make the participant feel

Family help: turning to family for support and help with childcare and emotional wellbeing

Favourable social environment: setting fosters interaction between the participant and others

Friendship: having or making friends

Like-community: having access to a social group with similar ethno-cultural practices, beliefs, and values as the participant

Other facilitators

Adequate time: time as a barrier to accomplishing tasks.

Attainable job requirements*:* requirements that need to be met by newcomers in order to find work in Canada.

Childcare: of or relating to caring for children i.e. education/daycare, looking after children, and spending time with them

Equitable laws and application of equitable laws: use of or impression of the laws in Canada and how they affect the lives and ideas of newcomers

Information/ advice/ counsel availability: finding/seeking information to solve a problem or orient oneself by asking for help or looking for information on a variety of topics from people and the internet

Interpretation/ translation: outcomes of situations based on the knowledge of or lack of knowledge of the local language (English in Toronto or French in Montréal).

Manageable climate conditions: relating to the weather in Canada

Permanent residence/ citizenship: Issues pertaining to an individual's immigration status in Canada, the reasons for immigrating, and consequences

Table 3

Interventions

Daycare: of or relating to formal care for pre-school children

Education: access to education for newcomers and their children; changes that could be made to the system regarding education

Employment: suggested changes for employment services and evaluation of current employment services in place

Food support: access to and availability of food for the participant's family

<u>Housing support</u>

General: general comments about provision and access to housing in Canada

Shelters: use and evaluation of shelters that provide refuge to those lacking essentials

YMCA: use and evaluation of the YMCA as a form of housing

Immigration procedures: challenges that occur during the process of applying for immigration and changes that should be made

<u>Health care</u>

Clinic: evaluation of the staff and services provided at clinics

Hospitals: relating to the use of and access to hospitals and health care professionals

Medical insurance: limitations of using Medicare (provincial health insurance)

Nurses: relating to the care provided by nurse (access, attitude, availability, satisfaction)

Physicians: relating to the care provided by doctors (access to doctors, attitude of the doctors, availability of the doctors, satisfaction of care provided)

<u>Organizations/programs</u>

Church: use of services and information provided by the church to newcomers

CLSC/ community health centre: Centre Local de Services Communautaires (CLSC) – information about the services provided (clinical and community) and the participants satisfaction

Family home visitor: evaluation and use of a family home visitor – a person who is introduced to the family by the home visit nurse and helps with everyday chores and activities (only in Toronto)

Mixed: use of multiple service centers and community organizations that newcomers can access for food, clothing, information, language, daycare and other things

OLO: use of the OLO (Orange-Lait-Œufs) program

PRAIDA: Programme régional d'accueil et d'intégration des demandeurs d'asile

SARIMM: use of the program -Service d'aide aux réfugiés et immigrants du Montréal Métropolitain (SARIMM)

<u>Psychosocial</u>

Social worker: satisfaction with the help provided by social workers

Psychologist: need for a psychologist

Social groups/activities: suggestions for new activities and groups to be created for newcomers; use of current social groups and activities

Welfare/ government assistance: relating to the money and/or support provided by the government through welfare and other services

Types of Service

Child care and development: relating to the well being of the participant's child

Information/ advice: obtaining information from other people (service providers and friends) about available services and how to access/use them

Maternal care: relating to the well being of the participant

Non-specified services: general comments and observations about interventions that were used

Provision of items: being given items or support from different community organizations including the church

Referral: getting directed to another person or place for more help and information

Teaching: obtaining knowledge and skills from trained professionals especially for child care

Quality of Service

Access: the ability to seek and use a service; having the service available for use

Affordability: having the financial means to access services provided such as daycare and medical care

Attitude: The behaviour of and manner in which service providers (health care professionals, social workers, daycare educators etc.) interact and treat the participant

Availability: presence of services, service providers, or locations for participants to access the resources needed to adapt to life in Montreal e.g. daycares, community centres, food banks etc.

Satisfaction: the participants evaluation of a service they have used or received

Table 5

Sites

CLSC/clinic: Any help received from a nurse at the CLSC or a clinic

Home: Any help received from a nurse in the participant's home

Hospital: Any help received from a nurse in the hospital

Info-Santé/ tele-health: Any help received from a nurse through Info-Sante (only in Montreal)

Types of Service

Assessment of baby: relating to the well being and development of the participant's baby

Information/advice: obtaining information from nurses about health care and available services and how to access/use them

Maternal care: relating to the well being of the participant

Referral: getting directed to another professional for more help

Teaching: obtaining knowledge and skills from nurses especially for child care

Quality of Service

Attitude: The behaviour of and manner in which nurses interact and treat the participant

Availability: presence and number of nurses for participants to see and access health care

Satisfaction: the participant's evaluation of the care and information received from nurses either in the hospital, at home, or at the clinic/CLSC
